# Oxidized low-density lipoprotein stimulates dendritic cells maturation via LOX-1-mediated MAPK/NF-κB pathway

**DOI:** 10.1590/1414-431X2021e11062

**Published:** 2021-05-31

**Authors:** D. Huang, W. Gao, H. Lu, J.Y. Qian, J.B. Ge

**Affiliations:** 1 Shanghai Institute of Cardiovascular Diseases, Zhongshan Hospital, Fudan University, Shanghai, P.R. China

**Keywords:** Lectin-like oxidized low-density lipoprotein receptor-1, Atherosclerosis, Dendritic cells, MAPK/NF-κB pathway

## Abstract

Dendritic cells (DCs) play a crucial role as central orchestrators of immune system response in atherosclerosis initiation and progression. Lectin-like oxidized low-density lipoprotein receptor-1 (LOX-1) is involved in the immune maturation of DCs, but the underlying mechanisms remain unclear. We isolated mouse bone marrow progenitors and stimulated them with granulocyte-macrophage colony-stimulating factor and interleukin (IL)-4 to induce immature DCs. We then treated DCs with oxidized low-density lipoprotein (oxLDL) to induce maturation. LOX-1 siRNA was used to investigate the modulation of LOX-1 on the development of DCs and the underlying signal pathways. CD11c-positive DCs were successfully derived from mouse bone marrow progenitors. OxLDL promoted the expressions of DCs maturation markers and pro-inflammatory cytokines. OxLDL also upregulated LOX-1 expression and activated MAPK/NF-κB pathways. LOX-1 siRNA could attenuate the expression of MAPK/NF-κB pathways and inflammatory cytokines. In conclusion, oxLDL induced the maturation of DCs via LOX-1-mediated MAPK/NF-κB pathway, which contributed to the initiation and progression of atherosclerosis.

## Introduction

Atherosclerotic vascular disease remains a leading worldwide cause of death and morbidity. Atherosclerosis is characterized as a chronic inflammatory disease of the vessel wall and is typified by an accumulation of lipids in arterial walls, the infiltration of many kinds of immune cells, and the formation of a fibrous cap (1). Accumulating evidence has suggested that dendritic cells (DCs) play a crucial role as central orchestrators of the immune response in atherosclerosis initiation and progression within arterial walls (2–5).

The uptake of oxidized low-density lipoprotein (oxLDL) through scavenger receptors by endothelial cells and immune cells is considered a critical step in the initiation and progression of atherosclerosis (6). Lectin-like oxidized low-density lipoprotein receptor-1 (LOX-1) can recognize both endogenous and exogenous ligands and play an essential role in the mediation of the effects of oxLDL on endothelial biology (7–9). Our group's previous work suggested that LOX-1 was involved in the dynamics of immune maturation of DCs stimulated with oxLDL and high glucose (10,11).

The LOX-1/p38 mitogen-activated protein kinase (MAPK) pathway has been proven to participate in endothelial dysfunction in atherosclerosis (12,13). However, the dynamics and mechanisms of the regulation of LOX-1 by oxLDL in DCs remain unclear. Thus, we sought to examine the effects of oxLDL upon the expression of LOX-1 in DCs and investigate the underlying mechanisms involved in this process.

## Material and Methods

### Dendritic cells culture and transfection

As described previously, bone marrow DCs were obtained from C57BL/6 mice (14). Briefly, the mice were sacrificed by cervical dislocation, and bone marrow progenitors were washed out from long bones (thigh and shin) and cultured in Iscove's modified Dulbecco's medium (IMDM, Gibco, USA) with 10% fetal bovine serum (FBS, Invitrogen, USA) containing 10 ng/mL granulocyte-macrophage colony-stimulating factor and 1 ng/mL interleukin (IL)-4 (PeproTech, USA). Non-adherent cells were gently washed out at 48 h. The remaining clusters, which were loosely adherent to the Petri dish, were cultured and the medium was changed every other day. On the 7th day, the cells were collected for treatment with different protocols depending on the subsequent studies.

For the immune maturation, DCs were treated with oxLDL (20 or 50 μg/mL; Meilun Technology, China) or PBS for 24 h.

For the transfection study, DCs were transfected with siRNA LOX-1 for 24 h and then treated with oxLDL or PBS for another 24 h. The DCs were then washed twice with PBS and replaced with fresh medium. The cells were then processed using a riboFECT CP transfection kit (Ribobio, China). LOX-1 siRNA was purchased from Ribobio, and the transfected concentration was 50 nM. The siR-Ribo Transfection Control (Cy3) was transfected into cells, and the results were observed using immunofluorescent microscopy (Zeiss, Germany). We used the siR-Ribo negative control during the transfection study.

### Flow cytometry

The expression of DCs surface marker was assessed by flow cytometry using cells stained with anti-CD80, anti-CD86, anti-CD40, anti-83, and anti-CD11c (BD Pharmingen, USA). Cells were washed twice, and immunofluorescence analysis was performed using FACScan flow cytometer (BD Biosciences, USA) and Cell Quest software (BD Biosciences).

### qRT-PCR

Total RNA was extracted from cells using TRIzol reagent (Sangon, China) following the manufacturer’s protocols and guidelines. ReverTra Ace qRT-PCR Kit (TOYOBO, Japan) was used to generate cDNA from mRNA. SYBR Premix Ex Taq (Takara, Japan) was used for qRT-PCR on the ABI 7500 Real-time PCR system platform following the manufacturer’s protocols and instructions. Primer sets for the amplification of mouse IL-1, IL-6, IL-10, IL-12, tumor necrosis factor (TNF)-α, and interferon (IFN)-γ are listed in Table 1.

**Table 1:** Primer sequence used in the study.

Gene	Primer	Sequence
IL-1	Forward	GCAACTGTTCCTGAACTCAACT
	Reverse	ATCTTTTGGGGTCCGTCAACT
IL-6	Forward	TAGTCCTTCCTACCCCAATTTCC
	Reverse	TTGGTCCTTAGCCACTCCTTC
IL-10	Forward	GCTCTTACTGACTGGCATGAG
	Reverse	CGCAGCTCTAGGAGCATGTG
IL-12	Forward	TGGTTTGCCATCGTTTTGCTG
	Reverse	ACAGGTGAGGTTCACTGTTTCT
TNF-α	Forward	CCCTCACACTCAGATCATCTTCT
	Reverse	GCTACGACGTGGGCTACAG
IFN-γ	Forward	ATGAACGCTACACACTGCATC
	Reverse	CCATCCTTTTGCCAGTTCCTC
β-actin	Forward	GGCTGTATTCCCCTCCATCG
	Reverse	CCAGTTGGTAACAATGCCATGT

### Western blotting

The isolated cells were lysed in RIPA buffer supplemented with complete protease inhibitor cocktail tablets (Roche, Switzerland). After 30 min of lysing, cell debris was removed by centrifugation at 10,800 *g* for 20 min at 4°C. Cell lysates were separated using SDS-PAGE gels. The constituents were transferred to PVDF membranes (Bio-Rad, USA), and the membranes were incubated with the relevant antibodies as indicated above. The relative intensities of protein bands were analyzed using Quantity One Software (BioRad, USA). All values were normalized to the GAPDH loading control. Anti-p-p38, c-fos, and p-p105 antibodies were purchased from Cell Signaling Technology (USA), and anti-LOX-1 was purchased from Abcam (USA).

### Statistical analysis

For quantification-based analyses, at least three independent replicates for each sample for each experiment were used. Data are reported as means±SD. Differences among groups were analyzed using one-away ANOVA followed by Fisher' exact test to compare two groups. All statistical analyses were performed with GraphPad Prism version 6 (USA). Differences between means were considered significant with P<0.05.

### Ethics statement

The study was approved by the Animal Care and Use Committee of Fudan University. All experimental procedures were performed following the Guide for the Care and Use of Laboratory Animals, published by the US National Institutes of Health.

## Results

### Dendritic cells culture and stimulation with oxLDL

**Figure 1:**
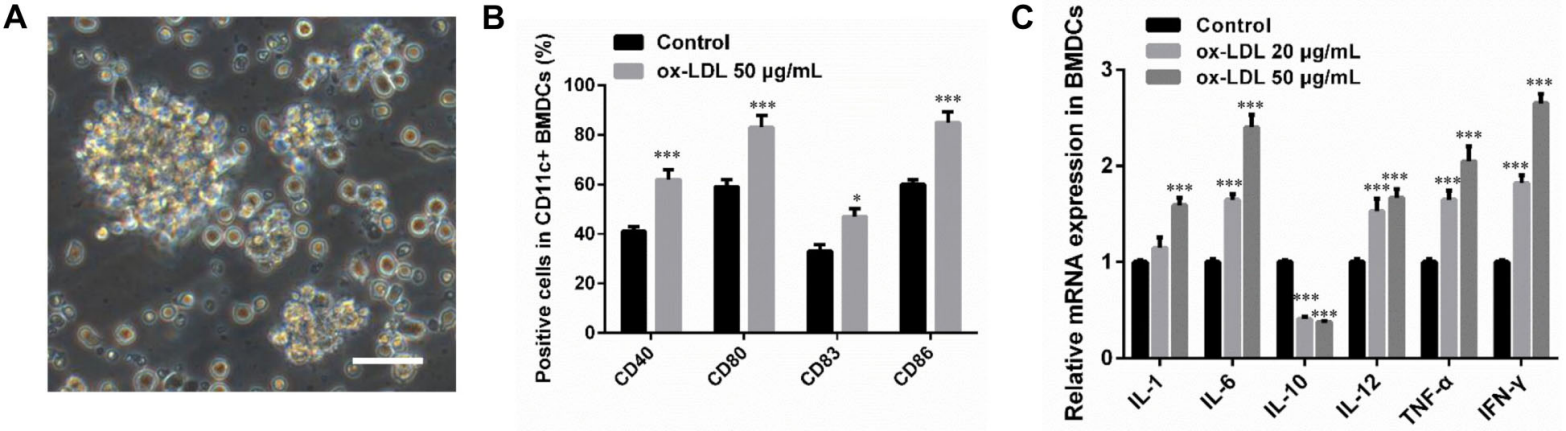
Bone marrow dendritic cells (BMDC) culture and stimulation with oxidized low-density lipoprotein (oxLDL). **A**, Typical morphology of BMDCs at day 7. Scale bar=200 μm. **B**, Statistical results of the surface markers of mature BMDCs by flow cytometry. **C**, qRT-PCR analysis of pro- and anti-inflammatory cytokines in BMDCs after treatment by oxLDL. Data are reported as means±SD (n=3). *P<0.05 *vs* control, ***P<0.01 *vs* control (ANOVA).

For the validity of IMDM in culturing bone marrow DCs, the cells were observed through a microscope and determined by the CD11c expression through flow cytometry on the 7th day. The results showed a satisfactory morphological structure (Figure 1A) and 85-90% of the cells were CD11c-positive.

In cultured bone marrow DCs, oxLDL treatment up-regulated DCs maturation markers such as CD40, CD80, CD83, and CD86 (Figure 1B), and increased pro-inflammatory cytokines, including TNF-a, IL-12, IL-1, IL-6, and IFN-γ (Figure 1C). However, the expression of the anti-inflammatory cytokine IL-10 was decreased (Figure 1C).

### OxLDL increased LOX-1 expression and activated MAPK/NF-&mac_kgr;B pathways

**Figure 2:**
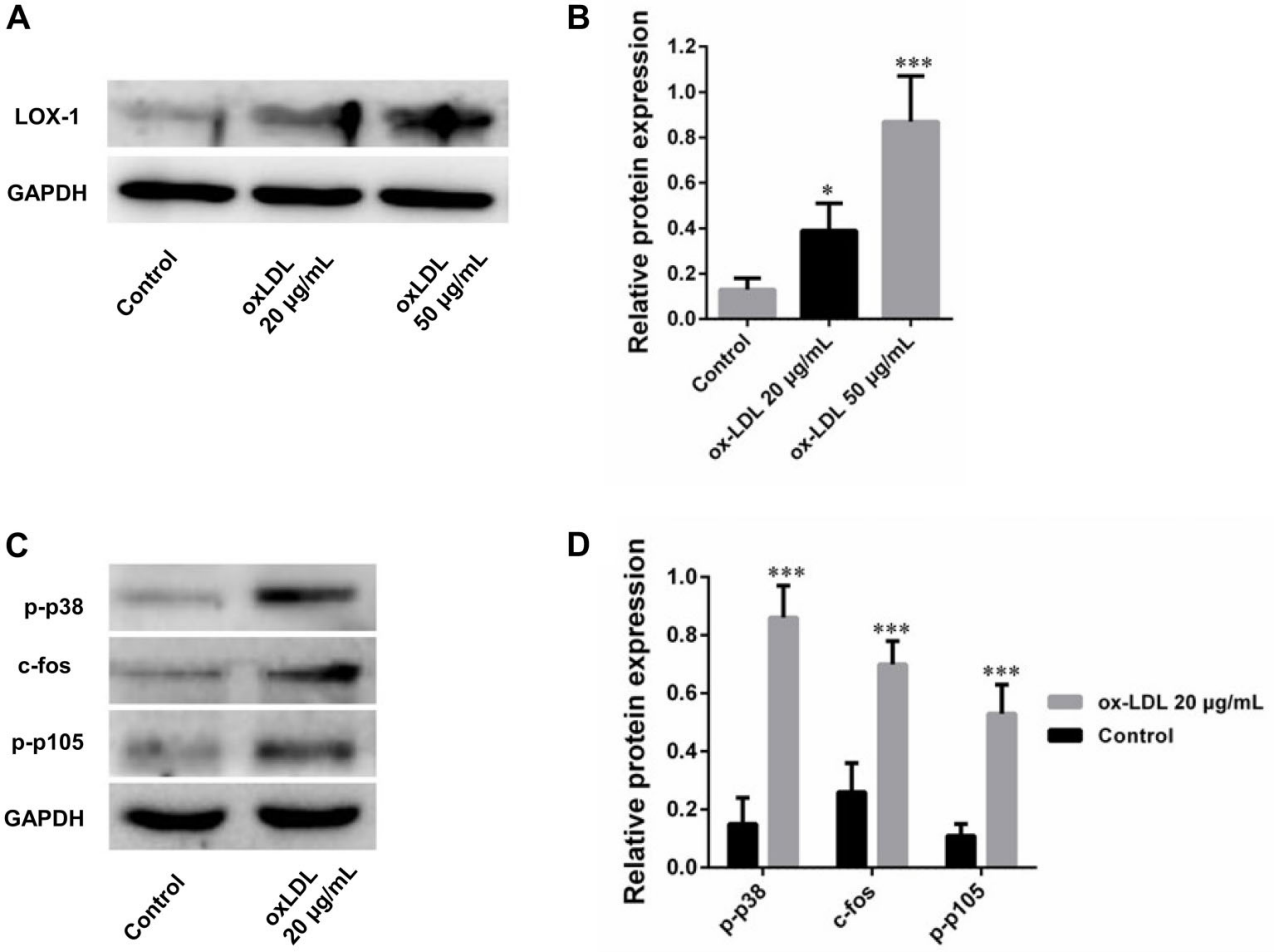
Lectin-like oxidized low-density lipoprotein receptor-1 (LOX-1) expression and MAPK/NF-κB pathway in dendritic cells (DCs) stimulated by oxidized low-density lipoprotein (oxLDL) (**A** and **B**). **C** and **D**, Expression of p-p38, c-fos, and p-p105 in DCs after treatment by oxLDL. Data are reported as means±SD (n=3). *P<0.05 *vs* control, ***P<0.01 *vs* control (ANOVA).

OxLDL upregulated LOX-1 expression at concentrations of 20 and 50 μg/mL (Figure 2A and B). The expressions of p-p38, c-fos, and p-p105 were increased in DCs stimulated with 20 μg/mL oxLDL (Figure 2C and D).

### LOX-1 expression was down-regulated by siRNA

**Figure 3:**
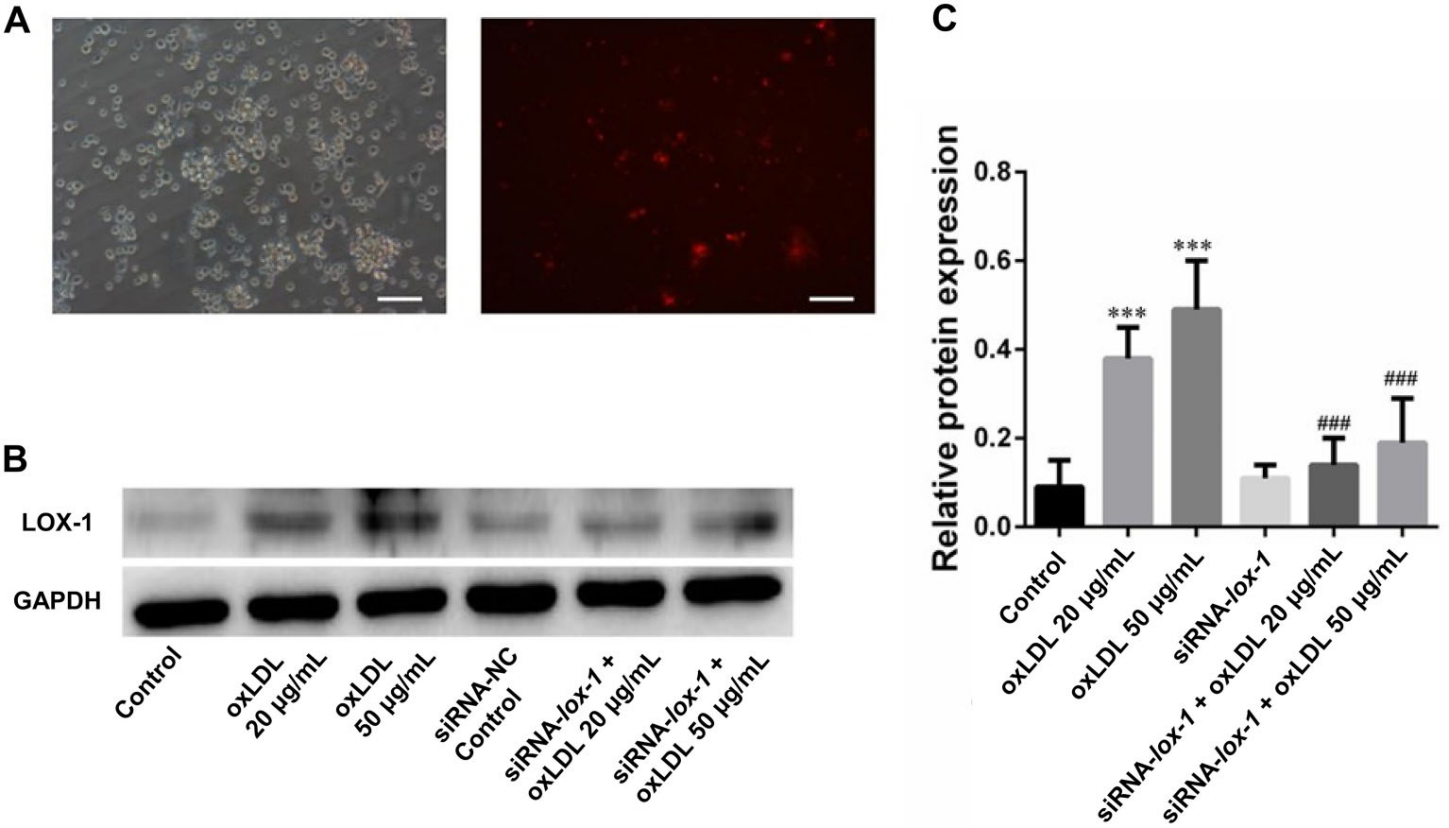
Dendritic cells (DCs) transfected with siRNA-LOX-1 and LOX-1 expression. **A**, The fluorescence observation of DCs after transfection with siRNA-cy3. Scale bar=200 μm. **B** and **C**, Expression of LOX-1 in DCs after transfection with siRNA-LOX-1 and treatment by oxidized low-density lipoprotein (oxLDL). Data are reported as means±SD (n=3). ***P<0.01 *vs* control, ^###^P<0.01 *vs* un-transfected groups (ANOVA).

To investigate the role of LOX-1, we used siRNA to induce downregulation of the expression of LOX-1 in DCs. The transfected siRNA-cy3 into DCs confirmed the successful transfection through immunofluorescent microscopy (Figure 3A). The transfection of LOX-1 siRNA suppressed the oxLDL-induced up-regulation of LOX-1 (Figure 3B and C).

### Inhibition of LOX-1 attenuated MAPK/NF-&mac_kgr;B pathways and inflammatory cytokines

**Figure 4:**
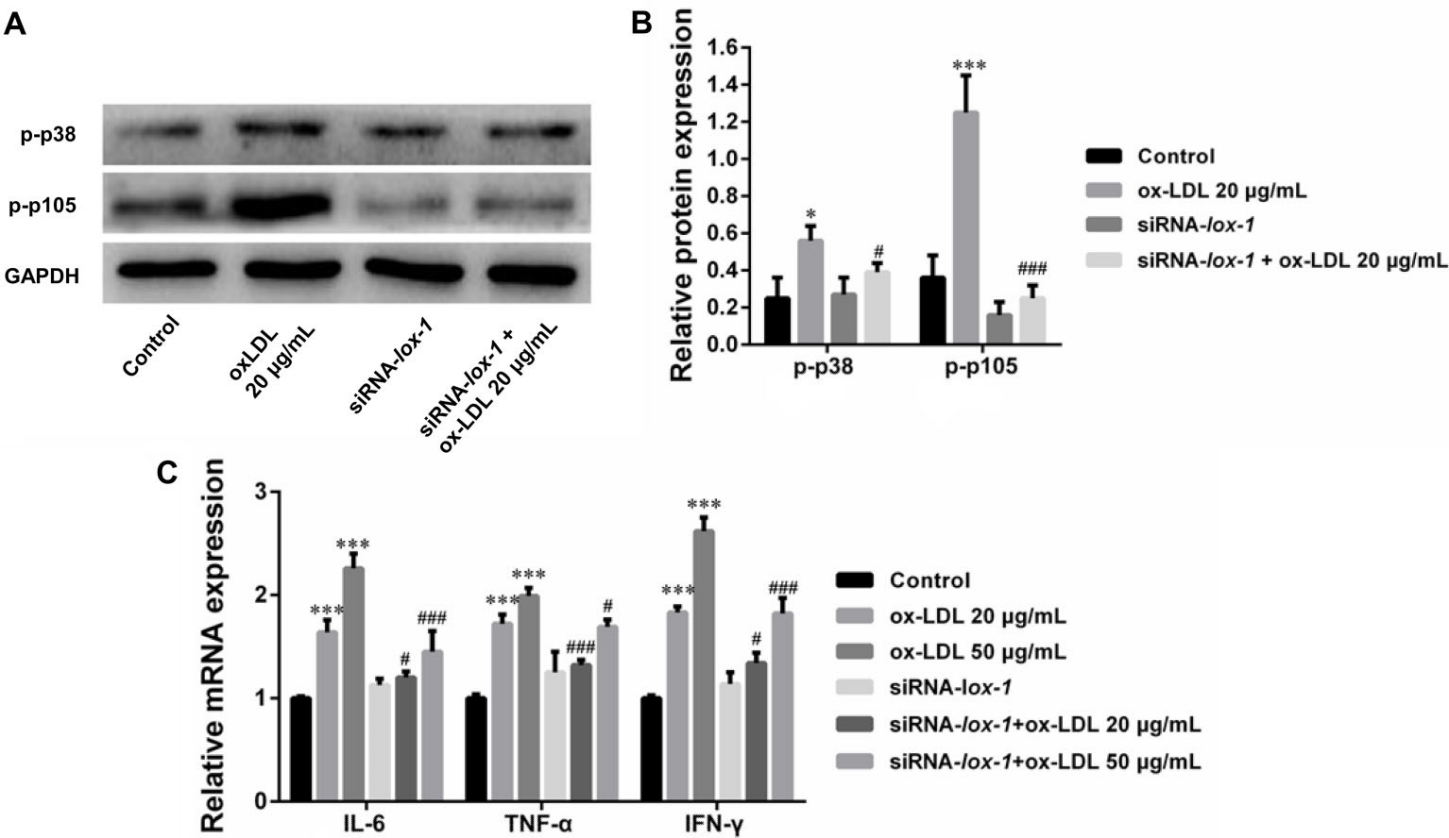
Inhibition of LOX-1 attenuated MAPK/NF-κB pathways and inflammatory cytokines. **A** and **B**, Expression of p-p38 and p-p105 in dendritic cells (DCs) after transfection with siRNA-LOX-1 and treatment by oxidized low-density lipoprotein (oxLDL). **C**, qRT-PCR analysis of pro-inflammatory cytokines in DCs after transfection with siRNA-LOX-1 and treatment by oxLDL. Data are reported as means±SD (n=3). *P<0.05, ***P<0.01 *vs* control; ^#^P<0.05, ^###^P<0.01 *vs* un-transfected groups (ANOVA).

Finally, we examined the role of LOX-1 in the regulation of MAPK/NF-κB pathways and inflammatory cytokines. The results showed that the inhibition of LOX-1 decreased the expression of p-p38 and p-p105 (Figure 4A and B). Subsequently, the transfection of LOX-1 siRNA suppressed the oxLDL-induced up-regulation of inflammatory cytokines, including IL-6, TNF-α, and IFN-γ, in DCs (Figure 4C).

## Discussion

In this study, we found that oxLDL stimulated the maturation of DCs via the LOX-1-mediated MAPK/NF-κB pathway. These findings could help better understand how immature DCs in vascular intima are activated by oxLDL and contribute to atherosclerosis initiation.

In mice, CD11c+ DCs are frequently located in the aortic intima in areas predisposed to atherosclerosis. DCs can also be detected in healthy young individuals' arterial intima, and increased numbers of DCs are found in atherosclerotic lesions (2,15). In the normal arterial intima, these resident DCs are thought to be immature (16). The maturation of DCs is the pivotal step for their function in the immune reaction. A variety of stimuli can initiate the maturation of DCs such as pathogens (lipopolysaccharides, bacterial DNA) and cytokines (17). Our study showed that oxLDL induced the maturation of DCs, accompanied with increased phenotypical expressions of maturation markers (CD40, CD80, CD83, and CD86) and pro-inflammatory cytokine secretions (TNF-a, IL-12, IL-1, IL-6, and IFN-γ), which was in agreement with the results of Zaguri et al. (18). Furthermore, the secretion of the anti-inflammatory cytokine IL-10 was decreased. Cytokines involved in human atherosclerosis can be broadly classified as pro-inflammatory and pro-atherogenic (such as IL-1, IL-6, and TNF) or as anti-inflammatory and anti-atherogenic (such as IL-10 and IL-1rA) (19). Perrin-Cocon et al. also reported that addition of oxLDL during the late stage of monocyte differentiation gives rise directly to phenotypically mature DCs, secreting IL-12 but not IL-10 (20). Induction of hypercholesterolemia in mice triggers rapid ingestion of lipids by resident intimal DCs, which initiate nascent foam cell lesion formation (21).

OxLDL uptake is mediated by the scavenger-receptors (22). LOX-1 is the dominant scavenger receptor that recognizes and internalizes oxLDL in endothelial cells and has also been found to be expressed on the surface of smooth muscle cells, platelets, fibroblasts, DCs, B cells, and macrophages (9). The LOX-1-oxLDL interaction induces endothelial dysfunction, leukocyte adhesion, macrophage-derived foam cell formation, smooth muscle cell proliferation and migration, and platelet activation (7). Our previous work suggested that LOX-1 is involved in the dynamics of immune maturation of DCs stimulated with oxLDL, high glucose, and angiotensin II (10,11). In endothelial cells, oxLDL induces the activation of LOX-1, which is associated with the p38 MAPK pathway (23). An *in vivo* study also demonstrated that p38 MAPK phosphorylation is reduced in the LOX-1 knockout mice (24), but some studies indicate that the activation of NF-κB induces the increase of expression of LOX-1 in endothelial cells (7,8). Our studies indicated that oxLDL increased the expression of LOX-1 in DCs and that both the p38 MAPK pathway and the NF-κB pathway were involved in this process.

Nickel et al. found that oxLDL increases the expression of the scavenger-receptors CD205 and CD36 and induces a proinflammatory cytokine profile in human DCs leading to DC-maturation and differentiation, but the LOX-1 expression is not affected (22). In contrast, we found that oxLDL upregulated the expression of LOX-1 of bone marrow DCs in mice with a relatively higher concentration (20 and 50 μg/mL) than the 10 μg/mL used in the previous study. Both studies showed that the blockage of the LOX-1 could reduce the oxLDL uptake and DC-maturation.

The upregulation of LOX-1 promoted the uptake of oxLDL and foam cell formation. Of therapeutic relevance, several natural products and clinically used drugs, including aspirin (25), losartan (26), and even testosterone (27), have emerged as LOX-1 inhibitors that have antiatherosclerotic actions (7). Our studies showed that the inhibition of LOX-1 attenuated the maturation of DCs induced by different stimuli such as oxLDL, angiotensin II, and high glucose, and subsequently, reduced foam cells formation (10,11). All the studies highlighted the potential of LOX-1 as a promising target of therapy for atherosclerosis and related disorders.
